# Association of Pathological Complete Response with Long-Term Oncological Outcomes After Neoadjuvant Chemoradiotherapy for Locally Advanced Rectal Cancer: A Retrospective Analysis of 327 Patients †

**DOI:** 10.3390/medicina62091639

**Published:** 2026-08-27

**Authors:** Berk Sertoz, Kamil Erozkan, Recep Temel, Murat Sezak, Basak Doganavsargil, Osman Bozbiyik, Cemil Caliskan, Erhan Akgun

**Affiliations:** Colorectal Surgery Division, Department of General Surgery, Ege University Hospital, 35100 Izmir, Turkey

**Keywords:** locally advanced rectal cancer, pathological complete response, neoadjuvant chemoradiotherapy, mesorectal excision, overall survival, disease-free survival

## Abstract

*Background and Objectives:* While total neoadjuvant therapy (TNT) is increasingly adopted, conventionally fractionated neoadjuvant chemoradiotherapy (CRT) followed by surgery remains the standard approach for many patients with locally advanced rectal cancer (LARC). Tumor regression following neoadjuvant therapy varies, potentially influencing oncologic outcomes. This study aimed to evaluate the association between pathological complete response (pCR) and long-term overall survival (OS) and disease-free survival (DFS) in patients with LARC undergoing neoadjuvant therapy and surgery. *Materials and Methods:* This retrospective study included patients who received long-course radiotherapy with concurrent capecitabine, followed by curative-intent surgery between January 2006 and January 2017. Patients were stratified according to the presence or absence of pCR. The primary outcomes were 5-year OS and DFS; secondary outcomes included locoregional recurrence and distant metastasis rates. *Results:* Among 327 patients, 47 (14.4%) achieved pCR. Patients with pCR had significantly lower pretreatment clinical T stage (*p* = 0.033) and clinical N stage (*p* = 0.049) and were more frequently classified as clinical stage II (55.3% vs. 40.0%, *p* = 0.049). Long-term oncological analyses included 322 patients after the exclusion of five early postoperative deaths. Locoregional recurrence (6.4% vs. 9.8%, *p* = 0.59) and distant metastasis (10.6% vs. 20.0%, *p* = 0.15) were numerically less frequent in the pCR group but did not differ significantly. Five-year OS was 91.5% in the pCR group and 73.1% in the non-pCR group (log-rank *p* = 0.027), while 5-year DFS was 85.1% and 61.5%, respectively (log-rank *p* = 0.008). After adjustment for age, sex, tumor location, pretreatment clinical T and N stage, and the CRT-to-surgery interval, pCR remained associated with improved DFS (adjusted HR 0.542, 95% CI 0.296–0.994, *p* = 0.048). In contrast, the association between pCR and OS was attenuated and did not reach statistical significance (adjusted HR 0.575, 95% CI 0.305–1.083, *p* = 0.087). *Conclusion:* pCR following neoadjuvant therapy in patients with LARC is associated with favorable long-term oncological outcomes. After adjustment for baseline clinical characteristics, pCR remained associated with improved DFS, whereas its association with OS was attenuated and did not reach statistical significance. These findings support the prognostic relevance of pCR while emphasizing the contribution of baseline disease characteristics to long-term survival.

## 1. Introduction

Over the past decade, the management of LARC has undergone a significant paradigm shift. TNT, which typically involves the delivery of systemic chemotherapy followed by CRT before surgical resection, has emerged as a preferred strategy. Multiple international guidelines now recommend TNT as the standard of care, citing its potential to improve systemic disease control and increase rates of tumor regression. Despite these advances, the real-world adoption of TNT remains heterogeneous, and conventional neoadjuvant CRT followed by delayed surgery continues to be widely practiced in many institutions [1,2,3,4].

Neoadjuvant CRT alone has been shown to achieve meaningful tumor downstaging, with pCR reported in approximately 8–20% of patients [5,6,7,8]. Achieving pCR is generally considered a favorable prognostic indicator and has been associated with reduced recurrence and improved survival outcomes. However, several challenges remain. First, the likelihood of achieving pCR depends on a range of factors, including baseline tumor biology, clinical stage, tumor location, and treatment regimen [9,10,11,12,13]. Second, while early outcomes in patients with pCR appear promising, locoregional recurrence and distant metastases may still occur. Finally, many of the existing studies assessing the prognostic value of pCR are limited by small sample sizes, heterogeneous treatment protocols, and relatively short follow-up periods, which restrict the ability to fully characterize its long-term oncological implications [12].

Although the favorable prognostic association of pCR after neoadjuvant chemoradiotherapy has been demonstrated in previous pooled analyses, systematic reviews, and cohort studies [14,15,16], long-term real-world data from relatively homogeneous cohorts treated with conventional long-course CRT remain clinically informative. Such cohorts provide an opportunity to evaluate long-term oncological outcomes within a consistent treatment framework and may serve as a historical reference in the era of increasing adoption of total neoadjuvant therapy. Therefore, the present study aimed to evaluate the association between pCR and long-term OS and DFS in a relatively large single-center cohort of patients with LARC treated with conventional long-course CRT followed by surgery and extended follow-up. We hypothesized that patients achieving pCR would demonstrate superior long-term OS and DFS compared with those without pCR.

## 2. Materials and Methods

### 2.1. Study Design and Patient Selection

**Figure 1:**
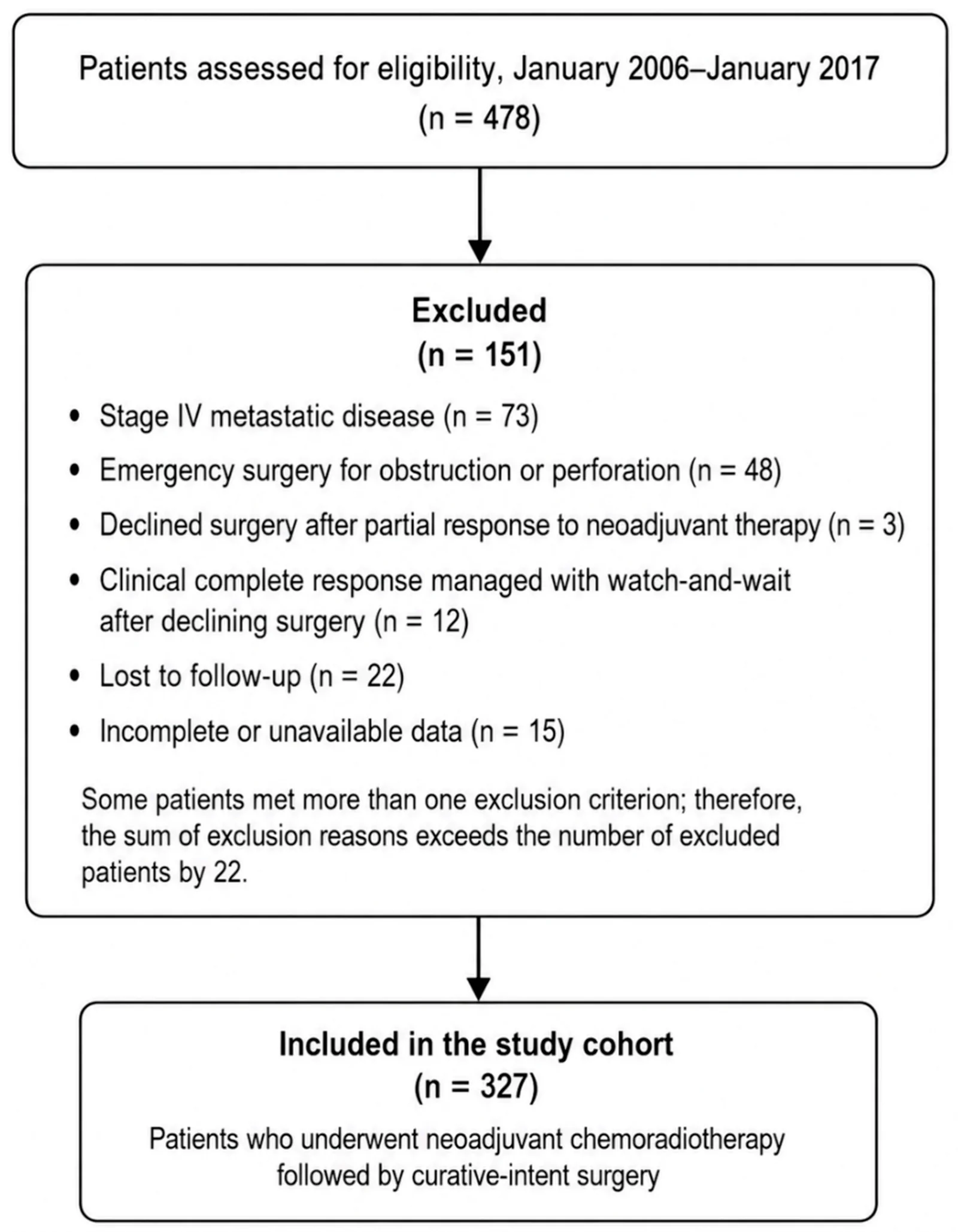
Patient selection flow diagram.

This retrospective cohort study encompassed patients diagnosed with locally advanced rectal adenocarcinoma who underwent neoadjuvant CRT followed by surgical intervention between January 2006 and January 2017 at a tertiary care center. Ethical approval was secured from the Institutional Review Board (Approval No.: 24-8T/86). Eligibility criteria included a diagnosis of locally advanced rectal adenocarcinoma within the specified timeframe, receipt of standard neoadjuvant CRT, and subsequent curative-intent rectal resection with mesorectal excision. Inclusion was limited to those with comprehensive clinical, radiological, pathological, and follow-up data. Tumor location was determined using rigid proctoscopy and available pelvic imaging. For the present analysis, tumors were categorized as lower rectal tumors, defined as those located within 5 cm of the anal verge, and mid-rectal tumors, defined as those located more than 5 cm and up to 10 cm from the anal verge. Exclusion criteria included stage IV metastatic disease, emergency surgery for obstruction or perforation, refusal of surgery after neoadjuvant treatment in patients with a partial response or no response, adoption of a watch-and-wait strategy in patients who achieved a clinical complete response (cCR) and declined surgery, loss to follow-up, and incomplete or unavailable clinical data (Figure 1).

### 2.2. Neoadjuvant Chemoradiotherapy and Restaging

Rectal cancer staging involved assessment through colonoscopy, available rectal magnetic resonance imaging (MRI), and computed tomography (CT) scans of the thorax and abdomen. Stage II (cT3/cT4 N0) and stage III (cN1/2) adenocarcinoma, commonly referred to as LARC, were identified based on these diagnostic findings. Treatment recommendations, including CRT, were determined by a multidisciplinary tumor board for all rectal cancer patients.

All patients received a standardized long-course CRT regimen, which comprised 50–50.4 Gy of pelvic radiotherapy administered in 25–28 fractions over 5.5 weeks, in conjunction with oral capecitabine at a dosage of 825 mg/m^2^ twice daily on radiotherapy days. Restaging was conducted four to six weeks following the completion of CRT, incorporating a digital rectal examination (DRE), flexible sigmoidoscopy, and MRI of the rectum to evaluate the local response and facilitate surgical planning. The MDTB evaluated the restaging results and reached a consensus on treatment recommendations. At the time of the study, radical rectal resection with appropriate mesorectal excision according to tumor level remained the standard recommendation after neoadjuvant CRT. Patients who achieved cCR but declined surgery and were managed with a watch-and-wait strategy were excluded from the final analytic cohort. Therefore, cCR was not analyzed as an endpoint in the present study. Patients were advised to undergo surgery within 6–12 weeks. Post-surgery, pCR was defined as the absence of viable tumor cells in both the primary tumor and regional lymph nodes (ypT0N0).

### 2.3. Surgical Technique, Adjuvant Chemotherapy and Follow-Up Protocol

Surgical interventions were conducted by experienced colorectal surgeons using an open approach. All patients underwent either low anterior resection (LAR) or abdominoperineal resection (APR). Mesorectal excision was tailored according to tumor level, with partial mesorectal excision (PME) performed in selected patients with mid-rectal tumors and total mesorectal excision (TME) performed for low rectal tumors and in patients requiring APR. The choice of surgical procedure and the extent of mesorectal excision were determined according to tumor location, its relationship to the anal sphincter complex, and the feasibility of achieving an oncologically safe distal margin. All patients were positioned in the modified lithotomy position under general anesthesia. A midline laparotomy was the incision of choice. Routine high ligation of the inferior mesenteric artery was performed. Mobilization of the splenic flexure was executed as necessary to achieve a tension-free anastomosis. In instances of suspected invasion of adjacent organs, en bloc resection was undertaken. Anastomoses were constructed using 28 or 32 mm circular staplers and were assessed for leakage via intraoperative air-leak testing and rectoscopy. A diverting ileostomy was created for anastomoses <5 cm from the anal verge, selectively considered for those 5–7 cm from the anal verge, and omitted for anastomoses >7 cm from the anal verge.

At our institution, adjuvant chemotherapy for patients with LARC following neoadjuvant CRT and curative resection is delivered in a patient-centered manner, most commonly with CAPOX or FOLFOX regimens. The CAPOX regimen consists of oxaliplatin 130 mg/m^2^ administered intravenously on day 1, combined with oral capecitabine 1000–1250 mg/m^2^ twice daily on days 1–14, repeated every 3 weeks for 6–8 cycles. The FOLFOX regimen is given every 2 weeks for 8–12 cycles and includes oxaliplatin 85 mg/m^2^ intravenously on day 1, leucovorin 400 mg/m^2^ intravenously, and 5-fluorouracil (5-FU) administered as a 400 mg/m^2^ bolus followed by a continuous infusion of 2400–3000 mg/m^2^ over 46 h. In accordance with patient-centered principles, the choice between CAPOX and FOLFOX, the number of cycles, and the timing of initiation (generally 4–8 weeks postoperatively) are tailored to individual patient factors such as performance status, comorbidities, organ function, tolerance to therapy, and patient preferences. Close monitoring is maintained throughout treatment to balance oncologic efficacy with quality of life and minimize toxicity. Patients classified as having completed treatment as planned had received all doses of the adjuvant chemotherapy regimen planned by the multidisciplinary team.

Postoperative monitoring encompassed physical examinations, CEA testing, thoracoabdominopelvic CT scans, and colonoscopy. Patients underwent evaluations every three months during the initial two years, every six months in years three to five, and annually thereafter. Colonoscopy was conducted at one year and subsequently every three years if no abnormalities were identified. Imaging intervals were modified based on the risk of recurrence.

Although the study covered an extended period from 2006 to 2017, the principal neoadjuvant treatment and surgical strategy remained relatively consistent, consisting of long-course capecitabine-based chemoradiotherapy followed by curative-intent resection. However, pelvic MRI quality and reporting evolved during this period, and contemporary MRI-based risk features were not consistently available, particularly in patients referred from outside institutions. Postoperative systemic treatment was individualized according to pathological findings, patient characteristics, treatment tolerance, and multidisciplinary assessment rather than being delivered according to a single uniform protocol throughout the study period.

### 2.4. Data Collection and Statistical Analysis

Patients were categorized into two cohorts based on pathological evaluation: those who achieved a pCR and those who did not. Clinical and pathological data were sourced from hospital archives and surgical pathology reports. Demographic information, including age, sex, and comorbidities, as well as tumor location, clinical staging, multivisceral resection, histologic subtype, pathological staging, resection margin status, lymphovascular invasion, perineural invasion, mesorectal excision quality, 30-day postoperative complications (Clavien–Dindo classification), adjuvant chemotherapy status, locoregional recurrence, distant metastasis, OS, and DFS were compared between the two groups. Postoperative complications were graded according to the Clavien–Dindo classification, and grade III or higher complications were considered major postoperative complications. DFS was defined as the interval from surgery to the date of the first occurrence of locoregional recurrence, metastasis, or death, whichever occurred first. Patients without reported recurrence or death at the time of analysis were censored at the last follow-up date. OS was defined as the interval from surgery to the date of death. Patients without reported death at the time of analysis were censored at the last follow-up date.

Data analysis was performed using IBM SPSS Statistics version 29.0 (Chicago, IL, USA). Continuous variables were evaluated for normality using the Shapiro–Wilk test. Student’s *t*-test or the Mann–Whitney U test was used for continuous variables. Chi-square tests were used for categorical variables. Fisher’s exact test was applied for 2 × 2 comparisons with small expected cell counts, whereas the Fisher–Freeman–Halton exact test was used for larger contingency tables when appropriate. Patients who died during the early postoperative period were excluded from the long-term oncological analyses. Survival outcomes were assessed using the Kaplan–Meier method and compared using the log-rank test. To account for potential confounding due to baseline differences between the pCR and non-pCR groups, multivariable Cox proportional hazards regression analyses were performed separately for OS and DFS. Covariates were selected a priori based on their clinical relevance and included pCR status, age, sex, tumor location, pretreatment clinical T stage, pretreatment clinical N stage, and the interval between completion of chemoradiotherapy and surgery. Tumor location was analyzed categorically as lower versus mid rectum. Clinical T and N stages were entered as categorical variables, with cT2 and cN0 serving as the reference categories, respectively. The interval between completion of chemoradiotherapy and surgery was categorized as ≤8 weeks or >8 weeks, with ≤8 weeks as the reference category. In an extended sensitivity analysis, mesorectal excision quality (complete vs. non-complete [near-complete/incomplete]) and adjuvant chemotherapy status (not administered, completed as planned, or incomplete/intolerant) were additionally incorporated into the baseline-adjusted Cox models. Adjusted hazard ratios (aHRs) with 95% confidence intervals (CIs) were calculated, and a two-sided *p*-value <0.05 was considered statistically significant.

## 3. Results

A total of 327 patients who underwent curative-intent resection for locally advanced rectal adenocarcinoma following neoadjuvant CRT were included. Among them, 47 patients (14.4%) achieved pCR, while 280 patients (85.6%) were classified as non-pCR. The demographic, clinical, histopathological, and long-term oncological characteristics of the pCR and non-pCR groups are summarized in Table 1. Patients in the pCR group had a slightly lower mean age compared with those in the non-pCR group (58.5 vs. 61.5 years, *p* = 0.10) and were more likely to present with lower rectal tumors (72.3% vs. 58.2%, *p* = 0.06), although these differences did not reach statistical significance. Sex distribution was comparable between the groups.

Significant differences were observed in pretreatment tumor stage. Patients who achieved pCR had lower pretreatment clinical T stage (*p* = 0.033) and N stage (*p* = 0.049). Consequently, pCR patients were more frequently diagnosed with stage II disease compared with the non-pCR group (55.3% vs. 40%, *p* = 0.049). Histopathological categories were comparable between the groups. The distribution of surgical procedures and the requirement for multivisceral resection also did not differ significantly between the pCR and non-pCR groups.

Postoperative pathological findings also differed according to treatment response. The mesorectal excision specimen was graded as complete more frequently in the pCR group than in the non-pCR group (97.9% vs. 88.2%, *p* = 0.044). Perineural invasion, lymphovascular invasion, and R1 resection margin positivity were not observed in patients with pCR and were present in 11.1%, 5.0%, and 9.7% of patients without pCR, respectively.

Major postoperative complications (Clavien–Dindo grade ≥III) occurred in 3 patients (6.4%) in the pCR group and 26 patients (9.3%) in the non-pCR group, with no significant difference between the groups (*p* = 0.78). Thirty-day postoperative mortality occurred in five patients (1.5%), all of whom were in the non-pCR group.

Adjuvant chemotherapy status was comparable between the pCR and non-pCR groups. Adjuvant chemotherapy was administered to 244 patients. Of these, 190 (77.9%) received FOLFOX and 54 (22.1%) received CAPOX. Overall, 206 patients (84.4%) completed all planned adjuvant chemotherapy doses, whereas 38 patients were classified as incomplete/intolerant. Among these 38 patients, the most frequently documented treatment-limiting toxicities were neutropenia in 14 patients (36.8%), peripheral neuropathy in 7 (18.4%), and hand–foot syndrome in 6 (15.8%). Adjuvant chemotherapy was not administered to 83 patients. Of these, 68 (81.9%) had stage II disease without additional high-risk features and did not receive postoperative chemotherapy following individualized multidisciplinary tumor board assessment. Among the remaining 15 patients, six declined chemotherapy despite the recommendation and nine were not considered suitable for treatment because of substantial comorbidities and concerns regarding treatment tolerance.

Long-term oncological analyses were performed in 322 evaluable patients after the exclusion of five patients who died during the early postoperative period. The median follow-up duration was 80 months. Locoregional recurrence occurred in 3 of 47 patients (6.4%) in the pCR group and 27 of 275 patients (9.8%) in the non-pCR group, with no statistically significant difference (*p* = 0.59). Similarly, distant metastasis occurred in 5 of 47 patients (10.6%) in the pCR group and 55 of 275 patients (20.0%) in the non-pCR group (*p* = 0.15).

**Figure 2:**
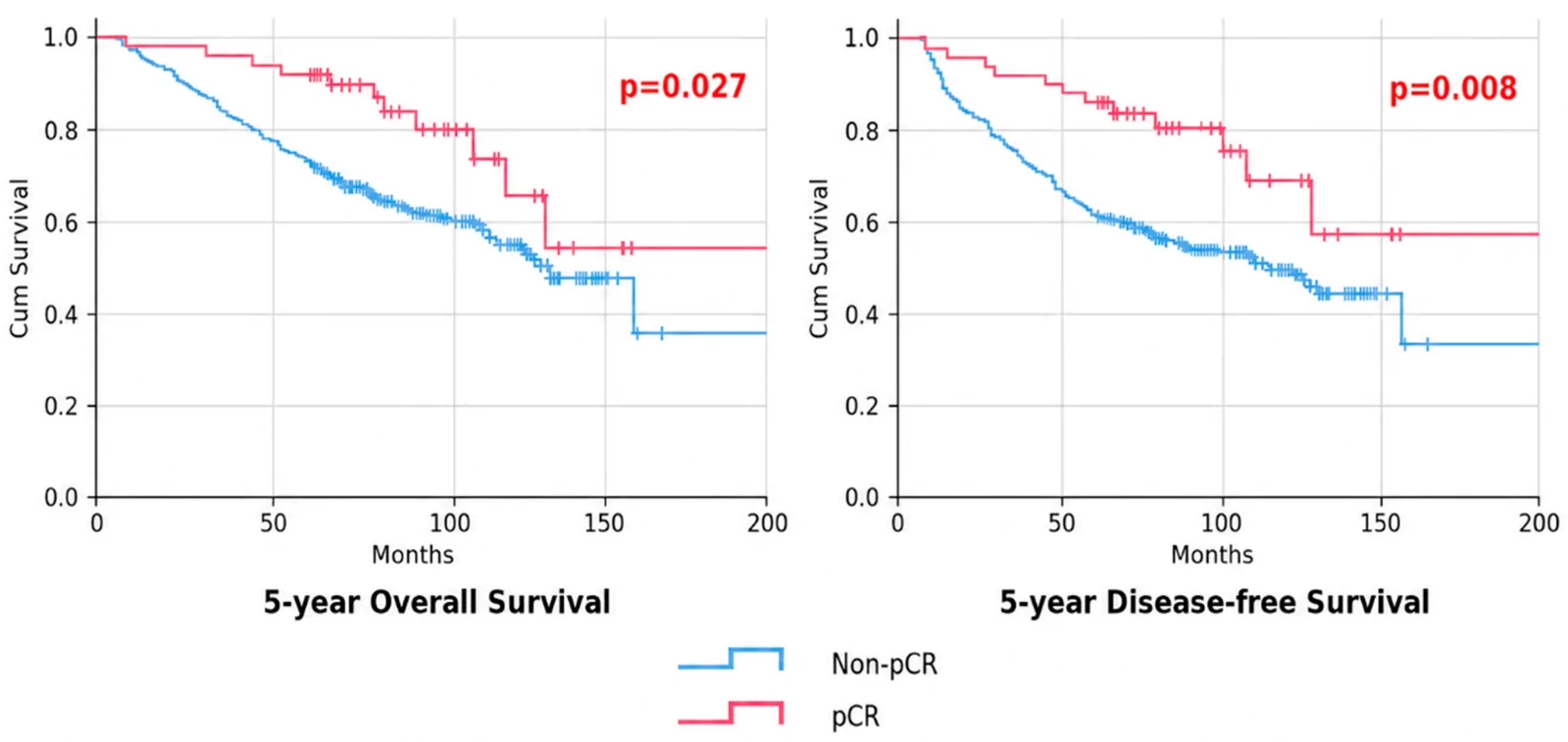
Overall and disease-free survival between patients with and without pathological complete response.

Kaplan–Meier analysis demonstrated superior unadjusted survival outcomes in patients who achieved pCR. Five-year OS was 91.5% in the pCR group compared with 73.1% in the non-pCR group (log-rank *p* = 0.027). Five-year DFS was 85.1% versus 61.5%, respectively (log-rank *p* = 0.008) (Figure 2). Consistent with these findings, univariable Cox proportional hazards analysis showed that pCR was associated with a significantly lower hazard of death (HR 0.504, 95% CI 0.271–0.935, *p* = 0.030) and a lower hazard of a DFS event (HR 0.456, 95% CI 0.253–0.824, *p* = 0.009).

To account for differences in baseline disease characteristics, multivariable Cox proportional hazards regression analyses were performed adjusting for age, sex, tumor location, pretreatment clinical T stage, pretreatment clinical N stage, and the interval between completion of chemoradiotherapy and surgery. After adjustment, pCR remained associated with improved DFS (aHR 0.542, 95% CI 0.296–0.994, *p* = 0.048). In contrast, the association between pCR and OS was attenuated and no longer reached statistical significance (aHR 0.575, 95% CI 0.305–1.083, *p* = 0.087). Increasing age and male sex were independently associated with worse OS and DFS, whereas tumor location, pretreatment clinical T stage, pretreatment clinical N stage, and the CRT-to-surgery interval were not independently associated with either survival endpoint (Table 2). In a sensitivity analysis additionally adjusting for mesorectal excision quality and adjuvant chemotherapy status, the association between pCR and both survival outcomes was further attenuated. In this extended model, pCR was not independently associated with OS (aHR 0.751, 95% CI 0.395–1.429, *p* = 0.383) or DFS (aHR 0.666, 95% CI 0.360–1.232, *p* = 0.195).

## 4. Discussion

This study demonstrated that pCR after neoadjuvant CRT was associated with favorable long-term oncological outcomes in patients with locally advanced rectal cancer. In the primary model adjusted for baseline clinical characteristics, pCR remained associated with improved DFS, whereas its association with OS was attenuated and did not reach statistical significance. However, in the sensitivity analysis additionally accounting for mesorectal excision quality and adjuvant chemotherapy status, the associations with both DFS and OS were further attenuated and were no longer statistically significant. These findings suggest that pCR is associated with a favorable prognosis, while also indicating that baseline disease characteristics, surgical quality, and postoperative treatment may contribute to the observed differences in long-term outcomes.

pCR following standard neoadjuvant chemoradiation and surgery in patients with LARC is consistently associated with improved long-term outcomes in pooled analyses, meta-analyses, and cohort studies [17,18,19]. Reported 5-year DFS rates in patients with pCR are commonly above 80%, with similarly favorable OS outcomes. In our study, unadjusted 5-year OS and DFS were also higher in the pCR group. Maas et al. reported a 5-year DFS rate of 83.3% in patients with pCR compared with 65.6% in patients without pCR [10], while de Campos-Lobato et al. reported improved overall survival and distant recurrence outcomes in patients achieving pCR [6]. These findings are consistent with our results. A comparison of representative studies evaluating long-term oncological outcomes according to pCR status is summarized in Table 3.

In our cohort, pCR was more frequently observed in patients with lower pretreatment clinical T and N stages, suggesting an association between lower baseline tumor burden and treatment response. Lower rectal tumors were also more frequent in the pCR group, although this difference was not statistically significant. These baseline imbalances are important because pretreatment disease burden may influence both treatment response and survival. After adjustment for age, sex, tumor location, clinical T stage, clinical N stage, and the interval between chemoradiotherapy and surgery, pCR remained significantly associated with improved DFS, with an approximately 46% reduction in the hazard of a DFS event. In contrast, its association with OS was attenuated and did not reach statistical significance. Perineural invasion, lymphovascular invasion, R1 resection margin status, and mesorectal excision quality were postoperative findings and were therefore interpreted descriptively rather than as baseline predictors of pCR. In particular, mesorectal excision quality cannot determine the biological response to neoadjuvant CRT; however, because mesorectal excision quality may independently influence local control and long-term oncological outcomes, the higher rate of specimens graded as complete for mesorectal excision quality in the pCR group may have contributed to the observed differences in survival.

In a sensitivity analysis additionally adjusting for mesorectal excision quality and adjuvant chemotherapy status, the association between pCR and both survival outcomes was further attenuated. In this extended model, pCR was not independently associated with either OS or DFS. The further attenuation of the association after accounting for mesorectal excision quality and postoperative systemic treatment suggests that differences in surgical quality and adjuvant treatment may partly contribute to the observed survival differences between the pCR and non-pCR groups. However, because these factors are determined after neoadjuvant treatment and may themselves be influenced by treatment response and postoperative pathological findings, these results should be interpreted as a sensitivity analysis rather than as an estimate of the independent causal effect of pCR.

Surgical procedure and the extent of mesorectal excision were similarly distributed between the pCR and non-pCR groups. Although the operative approach is clinically relevant to postoperative outcomes, the choice between LAR and APR and the extent of mesorectal excision are primarily determined by tumor location, anatomical considerations, and the feasibility of achieving an oncologically adequate resection. These postoperative surgical characteristics were therefore interpreted descriptively and were not considered predictors of pCR.

Although locoregional recurrence and distant metastasis were numerically less frequent in the pCR group, the differences were not statistically significant. Locoregional recurrence occurred in 6.4% of the pCR group and 9.8% of the non-pCR group, while distant metastasis occurred in 10.6% and 20.0%, respectively. Importantly, the absence of statistical significance should not be interpreted as evidence of equivalence between the groups, particularly given the approximately twofold higher distant metastasis rate in the non-pCR group. The small number of events, particularly in the pCR group, likely limited statistical power for these endpoints. Previous studies have similarly shown lower recurrence rates after pCR while demonstrating that recurrence risk is not completely eliminated [3,6,10]. These findings support continued postoperative surveillance even in patients who achieve pCR.

Results from major TNT trials, including RAPIDO [13], PRODIGE-23 [20], POLISH II [21], and STELLAR [22], have been inconsistent regarding the overall survival benefit. Although TNT has gained increasing use because of its potential to improve tumor regression compared with conventional CRT [23,24], its long-term survival advantage remains under investigation. Our cohort was treated with conventional long-course CRT followed by surgery; therefore, the present findings remain relevant to this treatment approach, although their applicability to contemporary TNT-treated populations should be interpreted with caution. The present study should therefore be viewed as complementary to, rather than fundamentally novel relative to, the existing evidence linking pCR with a favorable prognosis. Its principal contribution is the long-term evaluation of a relatively large single-center cohort treated within a predominantly consistent conventional long-course CRT framework. The extended follow-up provides additional real-world information on survival and recurrence patterns after this treatment approach and offers a historical reference against which outcomes from contemporary TNT-based strategies may be considered.

This study has several limitations that should be acknowledged. The retrospective and single-center design may introduce selection bias and limit the generalizability of the findings to broader populations. In addition, the extended treatment period from 2006 to 2017 may have introduced treatment-era effects related to changes in imaging quality, pathological assessment, perioperative care, and postoperative treatment practices that could not be fully accounted for in the present analysis. The exclusion of patients lost to follow-up may have introduced additional selection bias if their clinical characteristics or outcomes differed systematically from those of patients retained in the cohort. Additionally, many patients were referred from outside institutions, and contemporary MRI risk features were not consistently available, limiting detailed baseline risk adjustment. Adjuvant chemotherapy regimen and completion status were available, but exact cycle numbers, dose reductions, treatment delays, and cumulative dose intensity could not be reliably reconstructed for all patients. Although postoperative complications were recorded and graded according to the Clavien–Dindo classification, detailed information regarding specific complication types and their potential impact on adjuvant chemotherapy was not consistently available in the database. Furthermore, the absence of molecular and genetic profiling limits the ability to assess the influence of tumor biology on treatment response and survival. Although the overall cohort was relatively large, the pCR subgroup included only 47 patients, limiting statistical power and the precision of effect estimates, particularly for less frequent outcomes such as locoregional recurrence and distant metastasis. The relatively short follow-up period for some patients may also have resulted in an underestimation of late recurrences and long-term survival outcomes. Despite these limitations, the sizable cohort and extended follow-up provide useful long-term outcome data after conventional neoadjuvant CRT.

## 5. Conclusions

Pathological complete response following neoadjuvant chemoradiotherapy was associated with favorable long-term oncological outcomes in patients with LARC, including higher unadjusted 5-year OS and DFS rates. In the primary analysis adjusted for baseline clinical characteristics, the association between pCR and DFS persisted, whereas the association with OS was attenuated. However, additional adjustment for mesorectal excision quality and adjuvant chemotherapy status further attenuated these associations. These findings support an association between pCR and favorable long-term outcomes but should be interpreted cautiously given the retrospective design and potential influence of baseline and postoperative factors.

## Figures and Tables

**Table 1:** Baseline clinical, treatment-response, surgical and pathological, adjuvant treatment, and long-term oncological characteristics according to pathological complete response status.

Variable	All	pCR	Non-pCR	*p* Value
**BASELINE** **CLINICAL CHARACTERISTICS**				
**Age, years, mean ± SD**	61.0 ± 11.8	58.5 ± 11.6	61.5 ± 11.9	0.10
**Male sex,** ***n*** **(%)**	191 (58.4%)	28 (59.6%)	163 (58.2%)	0.86
**Tumor location,** ***n*** **(%)**				0.06
Mid rectal tumor	130 (39.8%)	13 (27.7%)	117 (41.8%)	
Lower rectal tumor	197 (60.2%)	34 (72.3%)	163 (58.2%)	
**Clinical T stage,** ***n*** **(%)**				**0.033**
cT2	12 (3.7%)	2 (4.3%)	10 (3.6%)	
cT3	205 (62.7%)	37 (78.7%)	168 (60.0%)	
cT4	110 (33.6%)	8 (17.0%)	102 (36.4%)	
**Clinical N stage,** ***n*** **(%)**				**0.049**
cN0	138 (42.2%)	26 (55.3%)	112 (40.0%)	
cN1	123 (37.6%)	17 (36.2%)	106 (37.9%)	
cN2	66 (20.2%)	4 (8.5%)	62 (22.1%)	
**Clinical stage,** ***n*** **(%)**				**0.049**
Stage II	138 (42.2%)	26 (55.3%)	112 (40.0%)	
Stage III	189 (57.8%)	21 (44.7%)	168 (60.0%)	
**Histopathological category,** ***n*** **(%)**				0.77
Well-differentiated	21 (6.4%)	2 (4.3%)	19 (6.8%)	
Moderately differentiated	265 (81.0%)	39 (83.0%)	226 (80.7%)	
Poorly differentiated	20 (6.1%)	2 (4.3%)	18 (6.4%)	
Mucinous adenocarcinoma	21 (6.4%)	4 (8.5%)	17 (6.1%)	
**TREATMENT-RESPONSE CHARACTERISTICS**				
**Pathological T stage,** ***n*** **(%)**				n/a
ypT0	48 (14.7%)	47 (100.0%)	1 (0.4%)	
ypT1	17 (5.2%)	-	17 (6.1%)	
ypT2	60 (18.3%)	-	60 (21.4%)	
ypT3	176 (53.8%)	-	176 (62.9%)	
ypT4	26 (8.0%)	-	26 (9.3%)	
**Pathological N stage,** ***n*** **(%)**				n/a
ypN0	222 (67.9%)	47 (100.0%)	175 (62.5%)	
ypN1a	35 (10.7%)	-	35 (12.5%)	
ypN1b	28 (8.6%)	-	28 (10.0%)	
ypN1c	7 (2.1%)	-	7 (2.5%)	
ypN2a	25 (7.6%)	-	25 (8.9%)	
ypN2b	10 (3.1%)	-	10 (3.6%)	
**Pathological stage,** ***n*** **(%)**				n/a
pCR	47 (14.4%)	47 (100.0%)	-	
Stage I	64 (19.6%)	-	64 (22.9%)	
Stage II	**111 (33.9%)**	-	111 (39.6%)	
Stage III	**105 (32.1%)**	-	105 (37.5%)	
**SURGICAL AND PATHOLOGICAL CHARACTERISTICS**				
**Surgical procedure,** ***n*** **(%)**				0.56 †
LAR with partial mesorectal excision (PME)	8 (2.4%)	0 (0%)	8 (2.9%)	
LAR with total mesorectal excision (TME)	197 (60.2%)	27 (57.4%)	170 (60.7%)	
APR with total mesorectal excision (TME)	122 (37.3%)	20 (42.6%)	102 (36.4%)	
**Multivisceral resection,** ***n*** **(%)**	20 (6.1%)	0 (0%)	20 (7.1%)	0.09
**Mesorectal excision quality,** ***n*** **(%)**				**0.044**
Complete	293 (89.6%)	46 (97.9%)	247 (88.2%)	
Near complete	21 (6.4%)	1 (2.1%)	20 (7.1%)	
Incomplete	13 (4.0%)	0	13 (4.6%)	
**Perineural invasion,** ***n*** **(%)**	31 (9.5%)	-	31 (11.1%)	n/a
**Lymphovascular invasion,** ***n*** **(%)**	14 (4.3%)	-	14 (5.0%)	n/a
**R1 resection margin,** ***n*** **(%)**	27 (8.3%)	-	27 (9.7%)	n/a
**Major complications (Clavien–Dindo ≥III),** ***n*** **(%)**	29 (8.9%)	3 (6.4%)	26 (9.3%)	0.78 †
**ADJUVANT CHEMOTHERAPY,** ***n*** **(%)**				0.11 †
Completed as planned	206 (63%)	36 (76.6%)	170 (60.7%)	
Incomplete/intolerant	38 (11.6%)	4 (8.5%)	34 (12.1%)	
Not administered	83 (25.4%)	7 (14.9%)	76 (27.1%)	
**LONG-TERM ONCOLOGICAL OUTCOMES**				
**Locoregional recurrence,** ***n*** **(%)**	30/322 (9.3%)	3/47 (6.4%)	27/275 (9.8%)	0.59 †
**Distant metastasis,** ***n*** **(%)**	60/322 (18.6%)	5/47 (10.6%)	55/275 (20.0%)	0.15 †
**5-year overall survival**	**75.8%**	**91.5%**	**73.1%**	**0.027 ‡**
**5-year disease-free survival**	**64.9%**	**85.1%**	**61.5%**	**0.008 ‡**

pCR: pathological complete response; SD: standard deviation; LAR: low anterior resection; APR: abdominoperineal resection; PME: partial mesorectal excision; TME: total mesorectal excision; CRT: chemoradiotherapy; OS: overall survival; DFS: disease-free survival. † Fisher’s exact test or the Fisher–Freeman–Halton exact test was used because of small expected cell counts. ‡ Kaplan–Meier estimates were compared using the log-rank test. Long-term oncological analyses included 322 evaluable patients (47 pCR and 275 non-pCR); five patients who died during the early postoperative period were excluded.

**Table 2:** Univariable and multivariable Cox proportional hazards regression analyses for overall and disease-free survival.

Variable	OS HR (95% CI)	*p* Value	DFS HR (95% CI)	*p* Value
**Univariable analysis**				
pCR vs. non-pCR	**0.50 (0.27–0.93)**	**0.030**	**0.45 (0.25–0.82)**	**0.009**
**Multivariable analysis**				
pCR vs. non-pCR	**0.57 (0.30–1.08)**	**0.087**	**0.54 (0.29–0.99)**	**0.048**
Age	1.03 (1.01–1.05)	<0.001	1.02 (1.01–1.04)	<0.001
Male vs. female sex	1.67 (1.14–2.44)	0.007	1.65 (1.16–2.36)	0.005
Lower vs. mid rectal tumor	1.38 (0.95–2.00)	0.08	1.38 (0.97–1.95)	0.06
cT3 vs. cT2	0.90 (0.33–2.47)	0.85	0.99 (0.37–2.67)	0.99
cT4 vs. cT2	1.72 (0.66–4.47)	0.26	2.04 (0.79–5.26)	0.13
cN1 vs. cN0	1.01 (0.62–1.59)	0.99	0.88 (0.56–1.38)	0.58
cN2 vs. cN0	1.20 (0.67–2.15)	0.53	1.20 (0.70–2.07)	0.50
CRT-to-surgery interval >8 vs. ≤8 weeks	0.88 (0.61–1.27)	0.51	0.78 (0.55–1.09)	0.15

HR, hazard ratio; CI, confidence interval; OS, overall survival; DFS, disease-free survival; pCR, pathological complete response; CRT, chemoradiotherapy.

**Table 3:** Comparison of representative studies evaluating long-term oncological outcomes according to pathological complete response status after neoadjuvant treatment for rectal cancer.

Study	Design	*n*	pCR/ Non-pCR, *n*	Follow-Up, Months	Main Oncological Findings
**de Campos-Lobato et al.** [6]	Single-center cohort	238	58/180	54	No locoregional recurrence occurred in the pCR group; overall survival and distant recurrence outcomes were significantly better in patients with pCR
**Maas et al.** [10]	Pooled individual-patient data analysis	3105	484/2621	48	5-year DFS: **83.3% vs. 65.6%**; HR for disease failure **0.44 (95% CI 0.34–0.57,** ***p*** **< 0.0001)**; adjusted HR **0.54 (95% CI 0.40–0.73)**
**Zorcolo et al.** [18]	Systematic review and meta-analysis (12 studies)	1913	300/1613	23–46 across studies	Locoregional recurrence: **0.7% vs. 2.6%**; distant recurrence: **5.3% vs. 24.1%**; 5-year OS: **92.9% vs. 73.4%**; 5-year DFS: **86.9% vs. 63.9%**
**Assaf et al.** [12]	Single-center retrospective cohort	263	53/210	60.7	5-year OS: **95.6% vs. 87.5% (*****p*** **= 0.09)**; 5-year DFS: **92.7% vs. 64.5% (*****p*** **< 0.001)**
**Present study**	Single-center retrospective cohort	327 *	47/275	80	5-year OS: 91.5% vs. 73.1% (*p* = 0.027); 5-year DFS: 85.1% vs. 61.5% (*p* = 0.008)

* Survival outcomes were calculated in 322 evaluable patients (47 pCR and 275 non-pCR) after the exclusion of five early postoperative deaths.

## Data Availability

The data supporting the findings of this study are available from the corresponding author upon reasonable request.
